# Novel *In Situ* Hybridization Assay for Chromogenic Single-Molecule Detection of Human Papillomavirus E6/E7 mRNA

**DOI:** 10.1128/spectrum.03896-22

**Published:** 2023-02-21

**Authors:** Xuelian Rao, Liangkai Zheng, Kaipeng Wei, Meiqing Li, Meng Jiang, Jianlong Qiu, Yulin Zhou, Rongqin Ke, Chen Lin

**Affiliations:** a School of Medicine, Huaqiao University, Xiamen, Fujian, China; b Department of Pathology, Women and Children’s Hospital, School of Medicine, Xiamen University, Xiamen, Fujian, China; c Department of Pathology, The 910th Hospital, Quanzhou, Fujian, China; d United Diagnostic and Research Center for Clinical Genetics, Women and Children’s Hospital, School of Medicine and School of Public Health, Xiamen University, Xiamen, Fujian, China; University of Manitoba

**Keywords:** human papillomavirus, padlock probe, RNA *in situ* hybridization, rolling circle amplification

## Abstract

RNA plays a vital role in the physiological and pathological processes of cells and tissues. However, RNA *in situ* hybridization applications in clinical diagnostics are still limited to a few examples. In this study, we developed a novel *in situ* hybridization assay for human papillomavirus (HPV) E6/E7 mRNA by taking advantage of specific padlock probing and rolling circle amplification, combined with chromogenic readout. We designed padlock probes for 14 types of high-risk HPV and demonstrated that E6/E7 mRNA could be visualized *in situ* as discrete dot-like signals using bright-field microscopy. Overall, the results are consistent with the clinical diagnostics lab’s hematoxylin and eosin (H&E) staining and p16 immunohistochemistry test results. Our work thus shows the potential applications of RNA *in situ* hybridization for clinical diagnostics using chromogenic single-molecule detection, offering an alternative technical option to the current commercially available kit based on branched DNA technology.

**IMPORTANCE** *In situ* detection of viral mRNA expression in tissue samples is of great value for pathological diagnosis to access viral infection status. Unfortunately, conventional RNA *in situ* hybridization assays lack sensitivity and specificity for clinical diagnostic purposes. Currently, the commercially available branched DNA technology-based single-molecule RNA *in situ* detection method offers satisfactory results. Here, we present our padlock probe- and rolling circle amplification-based RNA *in situ* hybridization assay for detecting HPV E6/E7 mRNA expression in formalin-fixed paraffin-embedded tissue sections, providing an alternative yet robust method for viral RNA *in situ* visualization that is also applicable to different types of diseases.

## INTRODUCTION

*In situ* DNA and protein biomarker analysis is widely used in clinical diagnostics, providing the histopathological status of diseases (1, 2). However, *in situ* detection of RNA biomarkers for clinical diagnosis purposes is limited to only a few examples, including kappa and lambda light chain mRNA *in situ* hybridization for clonality detection of mature B cell malignancies (3) and small Epstein-Barr virus-encoded RNAs (EBER) for assessment of Epstein-Barr virus (EBV) infection (4). Conventionally labeled cDNA or RNA probes constituted by natural or modified nucleic acids are used to hybridize with their target RNA sequences, allowing them to be visualized in cells or tissues (2). Over the past 2 decades, various single-molecule RNA *in situ* detection methods with improved sensitivity and specificity have been developed. Based on this, a series of imaging-based spatial transcriptomic techniques have also been introduced, such as *in situ* sequencing (ISS), STARmap, MERFISH, and seqFISH (5). Although notable advancements have been made in research, further efforts are still needed to translate these novel technologies into clinical diagnostics.

Cervical cancer (CC) as a result of cervical intraepithelial neoplasia (CIN) is the third most common female malignant tumor worldwide (6). Persistent human papillomavirus (HPV) infection is considered to be a cause of cervical intraepithelial lesions and invasive squamous cell carcinoma (SCC) (7). However, most HPV infections are transient and subclinical and can be cleared spontaneously by the immune system (8). But when the host gene is in a relatively unstable state because of damage caused by sexually transmitted diseases, chronic inflammation, or other chronic damage factors, the viral genome can be integrated into the host genome. HPV is manifested as a continuous infection state. At this time, E6/E7 mRNA is transcribed in large quantities, followed by the translation of E6/E7 proteins. E6/E7 proteins degrade p53 and inhibit pRB through interaction with other proteins, leading to the normal cycle of host cells out of control and eventually resulting in the development of cervical cancer (9). Thus, HPV E6/E7mRNA expression reflects the potential carcinogenicity of HPV, and its expression level is highly correlated with the severity of cervical lesions. Studies have shown that the positivity rate of HPV E6/E7 mRNA increases with the severity of cervical lesions (10). Therefore, *in situ* E6/E7 mRNA detection can offer valuable clinical information for assessing HPV infection status and its potential consequences.

**FIG 1:**
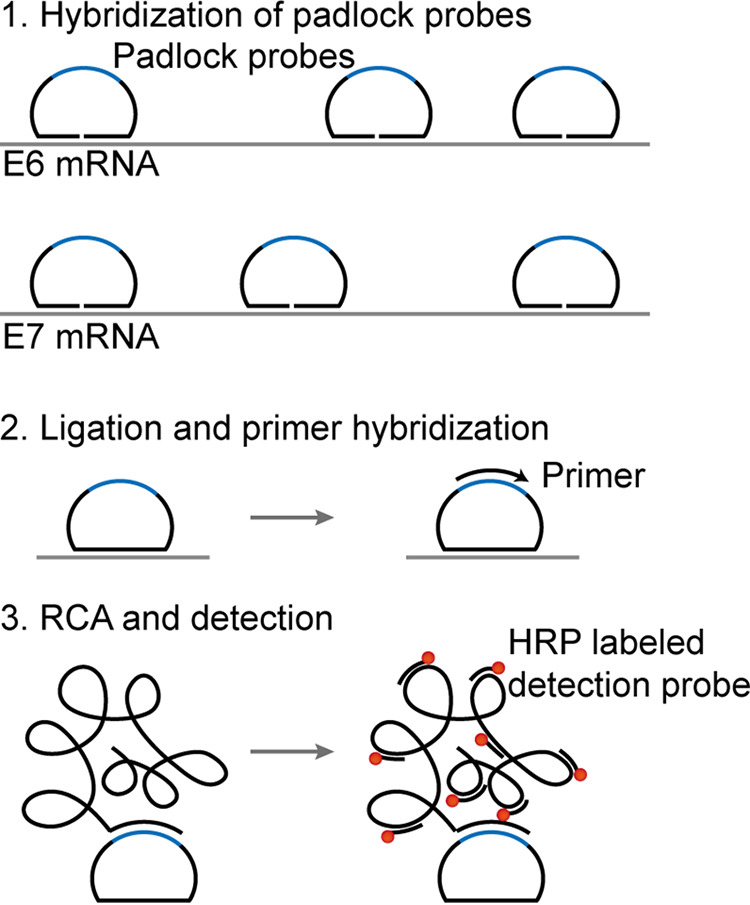
Schematic illustration of smCISH for HPV E6/E7 mRNA *in situ* detection.

Unfortunately, conventional methods for HPV E6/E7 mRNA *in situ* hybridization are not sensitive. Recently, RNAscope technology has been applied for *in situ* detection of HPV E6/E7 mRNA (11–13). RNAscope is a single-molecule RNA *in situ* hybridization assay that is based on branched DNA (bDNA) amplification technology, which can detect RNA expression sensitively and specifically at single-cell resolution (14). Similar to the conventional *in situ* hybridization methods, RNAscope can be read out by fluorescence or chromogenic detection. The advantage of chromogenic *in situ* hybridization (CISH) is that it allows simultaneous observation of tissue morphology and the target staining patterns. Furthermore, the stained samples can be stored for a more extended period and are in line with clinical diagnostic procedures. In this study, we used our previously developed single-molecule CISH (smCISH) technique (15) for detecting HPV E6/E7 mRNA expression. For our method, padlock probes targeting the E6/E7 mRNA of 14 high-risk HPV (HR-HPV) types were designed and applied. The padlock probes were all pooled; only the matching probe hybridized on its E6 or E7 mRNA targets, and the other unhybridized probes were then washed away. After hybridization, the padlock probes were circularized by DNA ligation on their mRNA templates using chlorella virus PBCV-1 DNA ligase that can ligate DNA probes using RNA as a template. After ligation, the padlock probes become circular DNA molecules and thus can be amplified by rolling circle amplification (RCA). The resulting rolling cycle amplification products (RCPs) are concatemers that contain hundreds of the original circles’ complementary sequences, which are then hybridized by horseradish peroxidase (HRP) enzyme-labeled DNA probes. The HRP-labeled RCPs can then be visualized by enzyme-catalyzed color development due to chromogen precipitation (Fig. 1). The RCPs appear as brown dot-like signals when observing them by bright-field microscopy, using 3,3′-diaminobenzidine (DAB) as the chromogenic substrate. Our method not only offers an alternative means of HPV E6/E7 mRNA *in situ* detection but also, for the first time, showcases the great potential of the *in situ* padlock probe assay for clinical diagnostic applications.

## RESULTS

### Validation of assay in cell line and formalin-fixed paraffin-embedded cervical biopsy sample.

**FIG 2:**
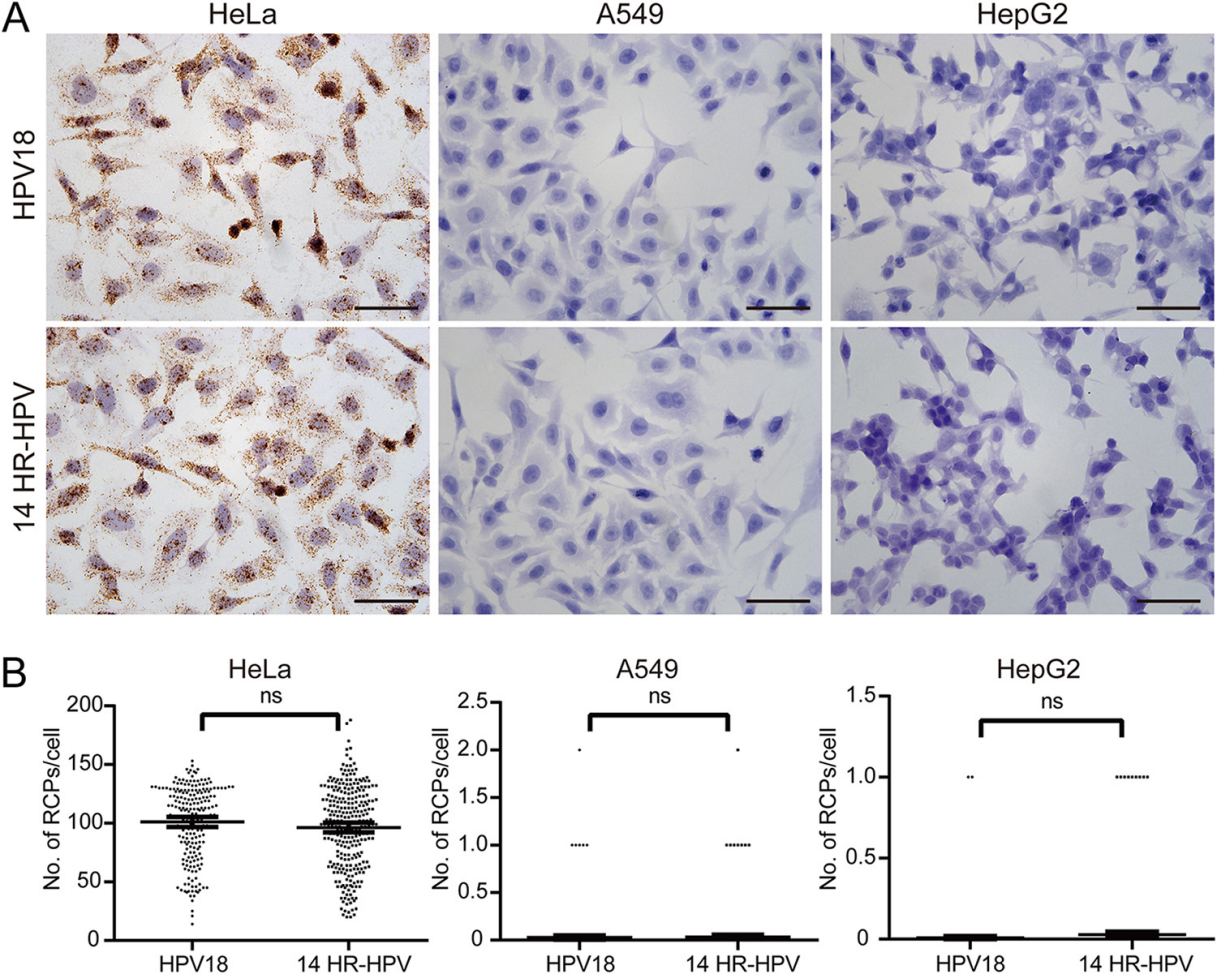
Detection of HPV E6/E7 mRNA using smCISH in cell culture with different probe combinations. (A) Images showing HPV E6/E7 mRNA signals in different cell types. Brown dots indicate DAB-stained E6/E7 mRNA probe RCPs resulting from smCISH. Nuclei are stained blue. Bars, 100 μm. (B) Statistical plots of the number of RCPs per cell from the images in panel A. ns, not significant.

*In situ* detection of HPV E6/E7 mRNA expression in the HeLa, A549, and HepG2 cell lines was used to validate the feasibility of our assay. Among these three cell lines, HeLa is a HPV-18-positive cell line derived from cervical carcinoma (16), while the A549 and HepG2 cell lines are HPV negative and originated from liver and lung cancer, respectively. To verify the feasibility and specificity of our assay, we performed our validation experiment either by using only padlock probes targeting E6/E7 mRNA for HPV-18 or a pool of padlock probes targeting all the E6/E7 mRNA from all the 14 high-risk HPV types separately. For the HeLa cells, the numbers of RCPs per cell detected by the HPV-18 probe and the 14 HR-HPV probe pool were 101.1 ± 2.160 (*n* = 210) and 96.31 ± 2.082 (*n* = 280), respectively. For A549 and HepG2, the numbers of RCPs per cell detected using the HPV-18 probe versus the 14 HR-HPV probe pool were 0.02682 ± 0.01140 (*n* = 261) versus 0.03180 ± 0.01159 (*n* = 283) and 0.0080 ± 0.005645 (*n* = 250) versus 0.02830 ± 0.009314 (*n* = 318), respectively (Fig. 2). These results showed that both probe sets gave rise to positive results for HPV E6/E7 mRNA in the HeLa cells and hardly any signal in either the HepG2 or A549 cells, suggesting our assay’s feasibility and excellent specificity. Statistical results showed that probes targeting either HPV-18 alone or all 14 types of HR-HPV have a nonsignificant difference in detection efficiency (*P* > 0.05), indicating that the competition and the increase of nonspecific signal of the pool of 14 HR-HPV probes are both minimal.

**FIG 3:**
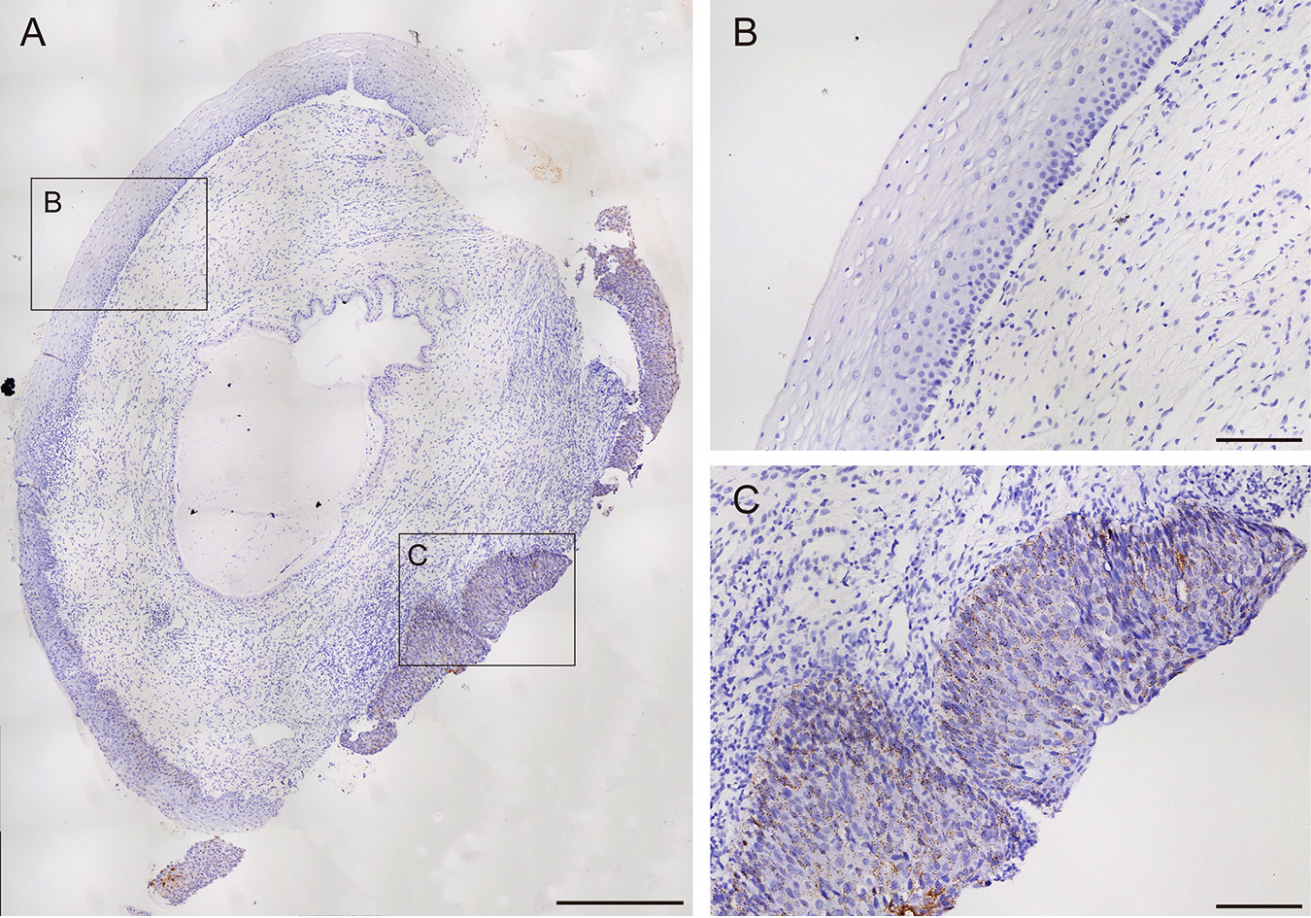
Validation of *in situ* HR-HPV E6/E7 mRNA detection in FFPE cervical biopsy specimen. (A) Overview of HPV E6/E7 mRNA detection in the whole biopsy specimen tissue. Bar, 500 μm. (B) Enlarged view of the LSIL region indicated in panel A. Bar, 100 μm. (C) Enlarged view of the HSIL region in panel A. Bar, 100 μm.

Next, we tested the feasibility of our method using a formalin-fixed paraffin-embedded (FFPE) cervical tissue biopsy specimen that tested positive for HPV DNA in its corresponding cervical swab sample. Padlock probes were used from here on for the E6/E7 mRNA of all 14 HR-HPV types unless otherwise stated. By scanning the whole tissue, we could see abundant dark brown dot-like signals from HPV E6/E7 mRNA in different regions of the epithelium. In contrast, there were almost no DAB staining signals or very few in the other areas (Fig. 3A). For example, there were hardly any DAB staining signals observed in the low-grade squamous intraepithelial lesion (LSIL) region (Fig. 3B). Still, substantially more dot-like DAB signals were observed in the high-grade squamous intraepithelial lesions (HSIL) regions (Fig. 3C). Furthermore, DAB signals were hardly seen in the other tissue regions apart from the epithelium, further demonstrating the specificity of our assay and its feasibility for application in real-life clinical samples.

### Validation of HPV E6/E7 mRNA detection in clinical samples.

To further validate that our method could detect HPV E6/E7 mRNA in clinical samples containing the 14 different HR-HPVs, we selected representative cervical biopsy specimens according to the cervical swab sample HPV DNA PCR test results provided by the clinical laboratory (Table 1). Model samples with single-type HPV infection were first enrolled for the test. At the same time, multiple infection specimens containing a specific type of HPV were also included if a single infection sample type was not found. HPV-45 was not validated because we could not find a corresponding infected specimen in the collected samples. We included an HPV-negative LSIL specimen as a negative control. Our HPV E6/E7 mRNA smCISH assay showed that all the PCR-positive specimens gave rise to positive E6/E7 mRNA staining, and no signals were observed in the negative samples (Table 1). These results thus validated the feasibility of our method for correctly detecting positive HPV infections, further showing its specificity and versatility. Images of E6/E7 staining in these clinical samples are presented in Fig. S1 in the supplemental material. In general, HPV E6/E7 mRNA staining appear as dot-like signals. Still, diffuse staining nuclei were observed in some instances due to the highly active transcription of HPV E6/E7 mRNA. These staining patterns are similar to previous findings in publications using the commercial RNAscope technology (11, 13). We further validated that we were detecting HPV E6/E7 mRNA but not DNA by performing a control experiment that used DNase I and RNase A to treat samples (Fig. S2). The results showed that after DNase treatment, there were no signals lost in the cell samples and visually, no signals lost in the tissue sample either. After treatment with RNase A, almost all RCPs were lost from the cell samples, but some nuclei still showed brown-stained signals, suggesting that HPV DNA might be detected by our assay. These findings are similar to those of previous publications that used the RNAscope assay to detect HPV E6/E7 mRNA (13). However, combining these results, we can conclude that the majority of signals being detected were from RNAs of the HPV E6 and E7 genes.

**TABLE 1:** Summary of tested clinical samples

Sample ID	Grade	HPV type(s)	p16 IHC	E6/E7 mRNA
1	HSIL	16	+	+
2	LSIL	18	+	+
3	HSIL	31	+	+
4	HSIL	33	+	+
5	HSIL	35, 52	+	+
6	LSIL	39	+	+
7	HSIL	16, 51	+	+
8	LSIL	52	+	+
9	LSIL	56	+	+
10	LSIL	58	+	+
11	LSIL	59	+	+
12	LSIL	51, 56, 66, 81	+	+
13	LSIL	68	+	+
14	NA^*a*^		−	−

a NA, not applicable.

### Comparison of E6/E7 mRNA staining patterns to H&E and p16 IHC staining patterns.

**FIG 4:**
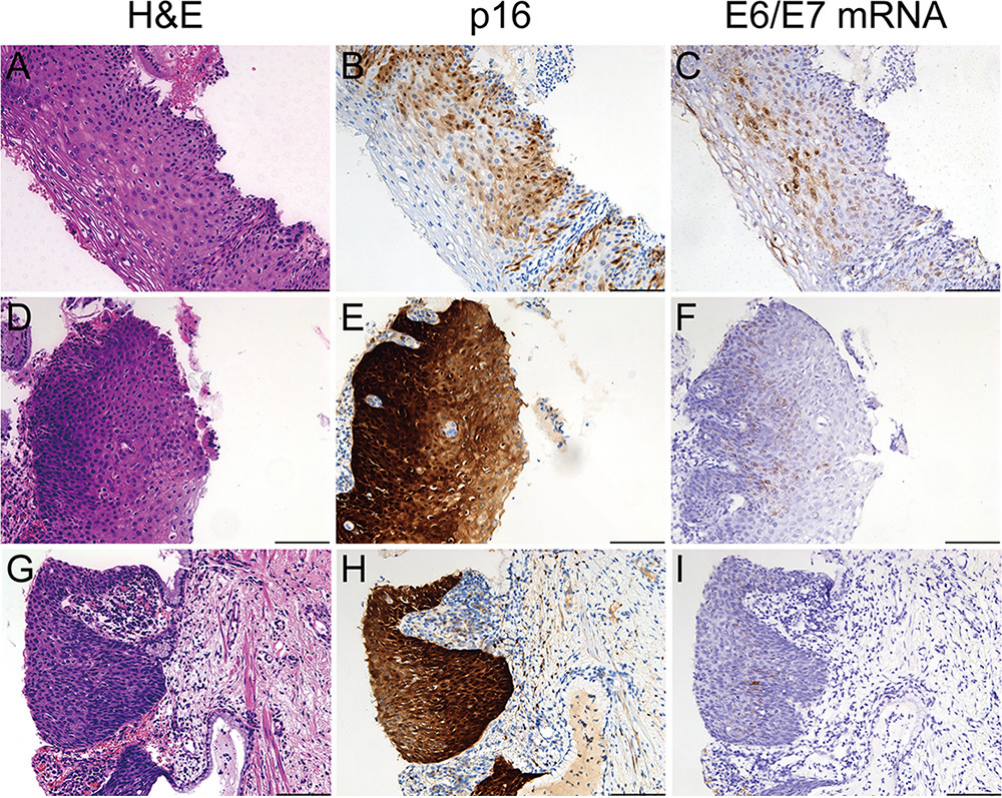
Comparison of H&E (A, D, and G), p16 IHC (B, E, and H), and HPV E6/E7 mRNA smCISH (C, F, and I) staining patterns of CIN1 (A to C), CIN2 (D to F), and CIN3 (G to I) specimens. Bars, 100 μm.

We compared HPV E6/E7 mRNA smCISH staining patterns to the hematoxylin and eosin (H&E) and p16 staining patterns in three samples, which were graded into LSIL (CIN1) or HSIL (CIN2 and CIN3) (Fig. 4). H&E staining indicated the abnormality of cells in the epithelium, and the p16 was positive for all three specimens. The p16 immunohistochemical (IHC) staining was mainly detected in the lower third of the epithelium in the LSIL specimens. In contrast, intense p16 IHC staining was observed throughout the lesions of the two HSIL specimens. HPV E6/E7 mRNA smCISH staining was positive for all three samples. At the same time, the LSIL specimen showed abundant diffusely stained nuclei in the upper epithelial layers. The two HSIL specimens showed multiple dot-like nuclear and cytoplasmic signals, mainly in the basal and lower epithelia. Notably, almost no E6/E7 mRNA staining signals were observed outside the lesions. These data again demonstrate that the RCA-based smCISH method is feasible for HPV E6/E7 mRNA *in situ* detection.

## DISCUSSION

This study presents a novel HR-HPV E6/E7 mRNA *in situ* hybridization assay that exploits our previously developed smCISH technology. The smCISH technique is a single-molecule RNA *in situ* detection method based on the padlock probe assay for specific target recognition and rolling circle amplification for signal enhancement combined with chromogenic readout (15). We first validated our assay by detecting HPV E6/E7 mRNA in known HPV-positive and -negative cell lines. The results showed that using a probe targeting only one type of HPV (HPV-18) or a pool of probes targeting 14 HR-HPV types could correctly detect positive and negative cells. In contrast, no signal was lost in the HPV-positive cells, and there was no increase in background fluorescence in the HPV-negative cells. We further validated our assay by detecting HPV E6/E7 mRNA in one cervical biopsy FFPE specimen. We observed that brown signal dots from the RCPs were only seen in the squamous epithelia or glands. Still, no signal dots were detected in the cell stromal, indicating that this method has excellent detection specificity. Next, we applied our method to more cervical biopsy specimens with different types of HR-HPV infections and one negative specimen, all tested by HPV DNA PCR assay using their corresponding cervical swab samples. The results showed that all the positive specimens gave rise to brown dot-like signals from HPV E6/E7 mRNA. In contrast, the negative specimens did not show any brown dot staining, demonstrating the feasibility and specificity of our normal HPV E6/E7 mRNA *in situ* hybridization assay. Comparison of our method to H&E staining and p16 IHC using specimens from three biopsies showed that most HPV E6/E7 mRNA signals were located in the lesion area. These results together showed that smCISH-based HPV E6/E7 mRNA ISH assay could correctly detect HPV infection in both cell and FFPE samples, demonstrating its feasibility for applications in clinical diagnosis.

Accurate assessment of cervical lesions is the key to managing and treating cervical intraepithelial neoplasia. A three-tier cervical intraepithelial neoplasia (CIN) approach, with grades 1, 2, or 3, was widely used initially. Subsequently, a two-tier approach that categorizes cervical lesions as low- or high-grade squamous intraepithelial lesions based on H&E staining and p16 immunohistochemistry was recommended (17). Because distinguishing low-grade from high-grade lesions can help avoid overtreatment, different biomarkers are being explored to better assist the grading of lesions. For example, a combination of biomarkers, such as p16 and *K_i_*-67 dual staining, could be used as an adjunctive test to improve the diagnostic accuracy of histopathology and detect CIN2+ among women with mild cervical lesions (18, 19). Adding HPV E4 immunohistochemistry to p16 and grading the expression of these two markers helps to identify the heterogeneity of HSILs (20). Minichromosome maintenance complexes (MCMs) and cell division cycle 6 (CDC6) proteins have also been used as biomarkers to predict the malignant potential of low-grade lesions (21). These studies suggest that accurate grading of cervical lesions may require combinations of different biomarkers. Our HPV E6/E7 mRNA detection assay can reflect the transcriptional status of HPV during infection, thus adding a new dimension to the pathological diagnosis of HPV infection. A systematic study that uses combinatorial detection of different biomarkers may benefit the diagnosis of cervical lesions in the future.

Analyzing DNA and protein biomarkers by *in situ* hybridization and immunohistochemistry is routine in clinical diagnostics. In contrast, *in situ* RNA detection is limited to a few examples, such as the EBER and kappa and lambda light chain mRNA *in situ* hybridization assays. Conventional RNA *in situ* detection has been challenging due to a lack of specificity and sensitivity. The commercial RNAscope technology, with its proprietary “double Z” probe design and bDNA technology for signal amplification, has been demonstrated to have overcome these issues (14). Use of RNAscope for different applications, such as HPV E6/E7 mRNA and albumin mRNA *in situ* hybridization, has shown its great potential for clinical diagnosis (13, 22). In this study, we demonstrated that our padlock probe and rolling circle amplification-based smCISH method could be used for clinically relevant HPV E6/E7 mRNA *in situ* detection, showing its great potential for clinical applications as an alternative approach. Although there are a series of novel RNA *in situ* detection technologies, such as single-molecule fluorescence *in situ* hybridization (smFISH), hybridization chain reaction (HCR), and so on, these technologies still lack application for real-life clinical diagnosis (5). As one of the novel RNA *in situ* detection methods, our method has been proven to work for detecting RNA molecules in FFPE samples, thus advancing the field of *in situ* biomarker analysis by opening up a new technical avenue. We believe that RNAs will become more widely used as clinical diagnostic biomarkers. To reach that end, standard protocols, such as sample preservation and pretreatments, have to be established for these kinds of *in situ* detection technologies. In conclusion, we have demonstrated that RCA-based RNA *in situ* hybridization is suitable for HPV E6/E7 mRNA detection in clinical samples. It can also be applied to other RNA molecule biomarkers for disease diagnosis.

## MATERIALS AND METHODS

### Cell samples.

A549 and HepG2 cells were cultured in high-glucose Dulbecco’s modified Eagle medium (DMEM) (Gibco) supplemented with 10% fetal bovine serum (FBS; Gibco) and 1% penicillin-streptomycin solution (Thermo Scientific). HeLa cells were cultured in MEM supplemented with 10% FBS and 1% penicillin-streptomycin solution. All cell lines were incubated at 37°C and 5% CO_2_. Cells were cultured in T flasks until confluent and then treated with 0.25% (wt/vol) trypsin-EDTA (Thermo Scientific). The resuspended cells were seeded onto Superfrost Plus microscope slides (Thermo Scientific) that were placed in a 150 mm by 25 mm petri dish (Corning); culture medium was then added to a final volume of 25 mL. The cells were again cultured under the same conditions overnight for attachment before fixation. After removal of the medium and two washes with diethyl pyrocarbonate treated with phosphate-buffered saline (DEPC-PBS), the cells were fixed in 4% (wt/vol) paraformaldehyde (PFA) (Sigma) in DEPC-PBS for 30 min and then washed twice with DEPC-PBS. Finally, dehydration of the cells was performed using a series of 70%, 85%, and absolute ethanol for 5 min each. These slides were then stored at −80°C before use.

### Tissue samples.

Formalin-fixed paraffin-embedded (FFPE) cervical biopsy specimens archived between 2021 and 2022 at Xiamen Women and Children’s Hospital were collected, and 4-μm sections were prepared for E6/E7 mRNA *in situ* hybridization analysis, as below. Related clinicopathological information of these samples was retrieved. The sections were stored at −80°C if not analyzed immediately. The ethical committee of the School of Medicine, Huaqiao University (M2021005), approved the use of the clinical samples.

### Sample pretreatment.

Pretreatment of the samples was performed as described previously (15). Briefly, cell samples were rinsed with 1× DEPC-PBS supplemented with 0.05% Tween 20 (DEPC-PBST) (Sigma) and then incubated in 0.1 M HCl for 10 min for permeabilization. For the FFPE samples, the tissues were fixed for a longer period of time and embedded in paraffin. Thus, the treatment was more complicated, requiring dewaxing and target retrieval using enzymatic digestion to be able to detect more mRNA signals. Briefly, tissue sections were first baked at 60°C for 30 min, and the samples were then submerged in xylene twice at room temperature (RT) for 15 min and 10 min to dewax. The slides were then dipped in 100%, 85%, and 70% ethanol, each twice for 2 min. The slides were then placed in DEPC-treated H_2_O (DEPC-H_2_O) for 5 min and washed with DEPC-PBS for 2 min. Next, fixation was carried out by adding 4% (wt/vol) PFA dropwise onto the slides to cover the tissue area and incubating them for 10 min at RT. The slides were then incubated in DEPC-PBS for 2 min. Immediately before permeabilization, pepsin (Sigma) was added to the preheated 0.1 M HCl to a final working concentration of 0.1 mg/mL pepsin. The slides were then submerged in the pepsin solution and permeabilized at 37°C for 30 min. Then, the slides were washed in DEPC-H_2_O for 5 min, followed by DEPC-PBS for 2 min. Dehydration was carried out by incubating the samples sequentially in 70%, 85%, and 100% ethanol, for 1 min at each concentration, and drying them at RT.

### Design of padlock probes for HPV E6 and E7 mRNA.

For our assay, we detected the E6/E7 mRNA of 14 high-risk HPV types, including HPV 16, 18, 31, 33, 35, 39, 45, 51, 52, 56, 58, 59, 66, and 68. The E6 and E7 mRNA sequences of the individual types of HPV were obtained from the National Center for Biotechnology Information database. We then used Primer3 (https://primer3.ut.ee/) to pick target sequences using the following rules (23). The primer sizes were set to be 15 to 18 nucleotides (nt), with 16 nt as the optimal length. The melting temperature (*T_m_*) of the primers was set to be between 45°C and 60°C, with a maximal *T_m_* difference of 5°C. The primer GC content was set to be between 40% and 60%. The product size was set to be between 32 and 34 nt, with an optimal size of 32 nt because the target hybridization sequence of the padlock probe was around 32 nt long. All the targets were subjected to a nucleotide BLAST search (https://blast.ncbi.nlm.nih.gov/Blast.cgi) to select the specific targets. We selected three target sequences for each E6 or E7 mRNA that distribute evenly on the mRNA, so as to avoid interference among the probes. The padlock probes were then designed using these targets so that their target hybridization arms were the complementary sequences to the target. Each arm was around 16 nt long, flanked by an interval sequence that contained the HRP-labeled probe hybridization site and other irrelevant sequences. The oligonucleotide sequences are listed in Table S1 in the supplemental material, and the target hybridization sites are highlighted in bold.

### Padlock probe hybridization and ligation.

An ImmEdge hydrophobic barrier pen (Vector Labs) was used in the following steps to create a hydrophobic barrier around the tissue sections to hold the reaction mix and wash solutions unless otherwise noted. The samples were washed three times with DEPC-PBS. The endogenous peroxidase activity was then blocked with 3% H_2_O_2_ by incubation at RT for 15 min, and the samples were washed three times with DEPC-PBS again. Next, a mixture containing 100 nM of each HR-HPV E6/E7 mRNA-targeting padlock probe (Table S1) in hybridization buffer (10% formamide in 6× SSC buffer [1× SSC is 0.15 M NaCl plus 0.015 M sodium citrate]) was added to the sample and incubated at 37°C for 2 h, followed by three washes with 2× SSC containing 20% formamide for 5 min each to remove the unhybridized padlock probes. Circularization of the padlock probes was carried out by adding a ligation mixture containing 1× SplintR ligase reaction buffer (NEB), 50% glycerol, 0.5 U/μL SplintR DNA ligase (NEB), 1 U/μL RNase inhibitor (Thermo Scientific), and 0.2 μg/μL bovine serum albumin (BSA; NEB) to the sample and incubating it for 0.5 h at 37°C. After washing the sample three times with DEPC-PBST, 50 μL of 200-nM RCA primers (Table S1) in hybridization buffer (6× SSC buffer and 10% formamide) was added to the sample, which was then incubated at 37°C for 30 min. The sample was again washed three times with DEPC-PBST.

### RCA and detection.

RCA was initiated by incubating the sample in a reaction mix containing 1× phi29 DNA polymerase reaction buffer (Thermo Scientific), 5% glycerol, 1 mM dNTPs (deoxynucleoside triphosphates; Thermo Scientific), 1 U/μL Equiphi29 polymerase (Thermo Scientific), and 0.2 μg/μL BSA in DEPC-H_2_O at 30°C for 16 h to 18 h and then washing the sample with DEPC-PBST three times. For RCA product (RCP) detection, 50 μL of probe mix containing 100-nM HRP-labeled detection probes (Table S1), 20% glycerol, 2× SSC, 0.05% (vol/vol) Tween 20, and 0.4 μg/μL BSA in DEPC-H_2_O was added to the sample, which was then incubated for 30 min at RT. After three washes with DEPC-PBST to remove the excess detection probes, 50 μL of DAB staining mixture (Sangon) containing 90% DAB buffer, 5% DAB substrate solution (20×), and 5% DAB chromogenic solution (20×) was added to the sample, which was then incubated for 3 to 8 min, depending on the color rendering. After washing the sample three times with DEPC-H_2_O, hematoxylin (Sigma) was used to stain the nuclei, followed by washing the sample three times with DEPC-H_2_O. The sample was incubated for 1 min with 0.1% hydrochloric acid in 70% ethanol for differentiation and washed three times with DEPC-H_2_O. Next, the slide was incubated in DEPC-PBS solution for 1 min and washed three times with DEPC-H_2_O. After rinsing with running tap water for 5 min, the slide was dried at RT and mounted with neutral balsam mounting medium (Sangon).

### Image acquisition and analysis.

Images were acquired using a Leica DM6B microscope equipped with a DFC7000T camera using a 20× objective. The RCPs appeared as dark brown dot-like spots. Quantification of RCPs in the cell samples was performed by analyzing three field of view (FOV) images from three slides using CellProfiler, as previously described (15). Unpaired *t* tests were used to compare data from the samples. At least 200 cells were analyzed per sample type. Morphologically, the normal epithelium in the FFPE block was used as an internal negative control.
