# Supplementary material for: Novel In Situ Hybridization Assay for Chromogenic Single-Molecule Detection of Human Papillomavirus E6/E7 mRNA
Source: Microbiol Spectr. 2023 Feb 21;11(2):e03896-22. doi: 10.1128/spectrum.03896-22 (PMC10101027; doi:10.1128/spectrum.03896-22)
Supplement: Supplemental file 1 — Supplemental material. Download spectrum.03896-22-s0001.pdf, PDF file, 2.7 MB [file spectrum.03896-22-s0001.pdf]

Supplemental Information

A novel in situ hybridization assay for HPV E6/E7 mRNA using chromogenic single molecule detection

Xuelian Rao [1,#], Liangkai Zheng [2,#], Kaipeng Wei [3,#], Meiqing Li [2], Meng Jiang [1], Jianlong Qiu [3], Yulin Zhou [4], Rongqin Ke [1,*], Chen Lin [1,*]

1. School of Medicine, Huaqiao University, Xiamen, Fujian, China
2. Department of Pathology, Women and Children's Hospital of Xiamen University, School of Medicine, Xiamen University, Xiamen, Fujian, China.
3. Department of Pathology, The 910th Hospital, Quanzhou, Fujian, China.
4. United Diagnostic and Research Center for Clinical Genetics, Women and Children's Hospital, School of Medicine & School of Public Health, Xiamen University, Xiamen, Fujian, China.

*Corresponding authors: rke@hqu.edu.cn (Rongqin Ke), chen.lin@hqu.edu.cn (Chen Lin)

[#] These authors contributed equally to the work.

Supplementary Table 1. Oligonucleotides

Names	Sequences (5'to3')	Functions
PLP-HPV16 E6-1	**TCTGTGCATAAATCTG**TTCCTTTTACGACCTCAATGCTGCTGCTGTACTACTCTT**TATAGTTGTTTGCAGC**	Padlock probe
PLP-HPV16 E6-2	**CATACAAACTATAACA**TTCCTTTTACGACCTCAATGCTGCTGCTGTACTACTCTT**TGTTCTAATGTTGTTC**	Padlock probe
PLP-HPV16 E6-3	**AAGACATACATCGACCG**TTCCTTTTACGACCTCAATGCTGCTGCTGTACTACTCTT**TCTTGATGATCTGCAAC**	Padlock probe
PLP-HPV16 E7-1	**AGTTGTCTCTGGTTGC**TTCCTTTTACGACCTCAATGCTGCTGCTGTACTACTCTT**CATAACAGTAGAGATC**	Padlock probe
PLP-HPV16 E7-2	**CTTGCAACAAAAGGTT**TTCCTTTTACGACCTCAATGCTGCTGCTGTACTACTCTT**GAAGCGTAGAGTCACA**	Padlock probe
PLP-HPV16 E7-3	**TGGACCATCTATTTCA**TTCCTTTTACGACCTCAATGCTGCTGCTGTACTACTCTT**GTTCTGCTTGTCCAGC**	Padlock probe
PLP-HPV18 E6-1	**ATGCAGCATGCGGTAT**TTCCTTTTACGACCTCAATGCTGCTGCTGTACTACTCTT**TCTATACATTTATGGC**	Padlock probe
PLP-HPV18 E6-2	**TCTATAGTGCCCAGCT**TTCCTTTTACGACCTCAATGCTGCTGCTGTACTACTCTT**ACGAATGGCACTGGCC**	Padlock probe
PLP-HPV18 E6-3	**TCCGTGCACAGATCAGG**TTCCTTTTACGACCTCAATGCTGCTGCTGTACTACTCTT**GCAGTGAAGTGTTCAGT**	Padlock probe
PLP-HPV18 E6-4	**ACCGCAGGCACCTTAT**TTCCTTTTACGACCTCAATGCTGCTGCTGTACTACTCTT**TTCAACGGTTTCTGGC**	Padlock probe
PLP-HPV18 E7-1	**TGCTGGGATGCACACC**TTCCTTTTACGACCTCAATGCTGCTGCTGTACTACTCTT**ATACAGGCACATTTAC**	Padlock probe
PLP-HPV18 E7-2	**TTGTGTTTCTCTGCGTC**TTCCTTTTACGACCTCAATGCTGCTGCTGTACTACTCTT**GGTCCATGCATTTATAC**	Padlock probe
PLP-HPV18 E7-3	**CTGGGATGCACACCAC**TTCCTTTTACGACCTCAATGCTGCTGCTGTACTACTCTT**ACAGGCACATTTACTG**	Padlock probe
PLP-HPV31 E7-1	**TCTTGCAATATGCGAA**TTCCTTTTACGACCTCAATGCTGCTGCTGTACTACTCTT**TGAGCCCATTAACAGC**	Padlock probe
PLP-HPV31 E7-2	**AGGTGTTTCTCCACGC**TTCCTTTTACGACCTCAATGCTGCTGCTGTACTACTCTT**CATAGTCTTGCAACGT**	Padlock probe
PLP-HPV33 E7-1	**CAATGTAGTAATCAGC**TTCCTTTTACGACCTCAATGCTGCTGCTGTACTACTCTT**GTGTGACAACAGGTTA**	Padlock probe
PLP-HPV33 E7-2	**CTGTGCCCATAAGTAG**TTCCTTTTACGACCTCAATGCTGCTGCTGTACTACTCTT**GGGCACACAATATTCA**	Padlock probe

PLP-HPV35 E6-1	**TACCTCGTTGCACAAA**TTCCTTTTACGACCTCAATGCTGCTGCTGTACTACTCTT**CATGGATGCTTTCTTC**	Padlock probe
PLP-HPV35 E6-2	**ATATACCTCACTCCGC**TTCCTTTTACGACCTCAATGCTGCTGCTGTACTACTCTT**CATAGCATGCAAAGTC**	Padlock probe
PLP-HPV35 E6-3	**TGGACACAGCGGTTTT**TTCCTTTTACGACCTCAATGCTGCTGCTGTACTACTCTT**GTCTTTGCTTTTCAAC**	Padlock probe
PLP-HPV35 E7-1	**AGACGTAGTGTCGCCTC**TTCCTTTTACGACCTCAATGCTGCTGCTGTACTACTCTT**GTGTGCTCTGTACACAC**	Padlock probe
PLP-HPV35 E7-2	**CAAATGTGCCCATTAA**TTCCTTTTACGACCTCAATGCTGCTGCTGTACTACTCTT**CCGGGGCACACTATTC**	Padlock probe
PLP-HPV39 E6-1	**AGACAACACATGCACC**TTCCTTTTACGACCTCAATGCTGCTGCTGTACTACTCTT**TGGACACAGCGGTTTC**	Padlock probe
PLP-HPV39 E6-2	**CGTCGACACTGTCCTG**TTCCTTTTACGACCTCAATGCTGCTGCTGTACTACTCTT**TTTTGTGGTCCAGCAC**	Padlock probe
PLP-HPV39 E7-1	**TGGTTTGCAGTTGCAC**TTCCTTTTACGACCTCAATGCTGCTGCTGTACTACTCTT**GGCCATAGCAGGTTAC**	Padlock probe
PLP-HPV39 E7-2	**TAATTGCTCGTGACAT**TTCCTTTTACGACCTCAATGCTGCTGCTGTACTACTCTT**CATCCTCTGACTCTCC**	Padlock probe
PLP-HPV39 E7-3	**TTGTGTGACGCTGTGGT**TTCCTTTTACGACCTCAATGCTGCTGCTGTACTACTCTT**ACAACACGAACACTGTA**	Padlock probe
PLP-HPV45 E6-1	**TGCGAAGTCTTTCTTG**TTCCTTTTACGACCTCAATGCTGCTGCTGTACTACTCTT**TGTGTTTCCCTACGTC**	Padlock probe
PLP-HPV45 E6-2	**TGGTAGCTTGTAGGGT**TTCCTTTTACGACCTCAATGCTGCTGCTGTACTACTCTT**ATTCTGTGCACAAATC**	Padlock probe
PLP-HPV45 E6-3	**TTCAATGGTTTCTGGC**TTCCTTTTACGACCTCAATGCTGCTGCTGTACTACTCTT**ACGTTTTTCTGCTGGG**	Padlock probe
PLP-HPV45 E7-1	**TAAGCTCAATTCTGCC**TTCCTTTTACGACCTCAATGCTGCTGCTGTACTACTCTT**GCCGAGCTCTCTACTG**	Padlock probe
PLP-HPV45 E7-2	**CAAATGCAATACAATTT**TTCCTTTTACGACCTCAATGCTGCTGCTGTACTACTCTT**AATTCATTCTGAGGTTC**	Padlock probe
PLP-HPV45 E7-3	**CATTACACCCCGTTCC**TTCCTTTTACGACCTCAATGCTGCTGCTGTACTACTCTT**TCTACAAAAAACCAGC**	Padlock probe
PLP-HPV51 E6-1	**ATAATTCATGCAGCGT**TTCCTTTTACGACCTCAATGCTGCTGCTGTACTACTCTT**ACGTTCAAAGCTTCAC**	Padlock probe
PLP-HPV51 E6-2	**ACGTTGCCAGCAATTA**TTCCTTTTACGACCTCAATGCTGCTGCTGTACTACTCTT**CGTTACGTTGTCGTGT**	Padlock probe
PLP-HPV51 E7-1	**TGTAACACGTAGCCTG**TTCCTTTTACGACCTCAATGCT	Padlock probe

	GCTGCTGTACTACTCTT**CACGGAGCTTCAATTC**	
PLP-HPV51 E7-2	**ACAACACGGGCAAACC**TTCCTTTTACGACCTCAATGCT<br>GCTGCTGTACTACTCTT**GTTGCTAGTTGTTCGC**	Padlock probe
PLP-HPV52 E6-1	**AATTCGTGCAGGGTCC**TTCCTTTTACGACCTCAATGCT<br>GCTGCTGTACTACTCTT**TTCCAGCACCTCACAC**	Padlock probe
PLP-HPV52 E6-2	**CATATGGATTATTGTC**TTCCTTTTACGACCTCAATGCTG<br>CTGCTGTACTACTCTT**CACATAATACACACGC**	Padlock probe
PLP-HPV52 E6-3	**CACACGCCATATGGAT**TTCCTTTTACGACCTCAATGCTG<br>CTGCTGTACTACTCTT**GCGTAGGCACATAATA**	Padlock probe
PLP-HPV52 E6-4	**TCAGGACATAATGGCGT**TTCCTTTTACGACCTCAATGC<br>TGCTGCTGTACTACTCTT**CATGTCTTTCTTTTTCC**	Padlock probe
PLP-HPV52 E7-1	**TCATAGCAGTGTAGGT**TTCCTTTTACGACCTCAATGCT<br>GCTGCTGTACTACTCTT**GCTGTCACCTAATTGC**	Padlock probe
PLP-HPV52 E7-2	**CGGTCCACACCATCTG**TTCCTTTTACGACCTCAATGCT<br>GCTGCTGTACTACTCTT**TGCTTGTCCATCTGGC**	Padlock probe
PLP-HPV52 E7-3	**TATGAATGCATAGCCG**TTCCTTTTACGACCTCAATGCTG<br>CTGCTGTACTACTCTT**AGGTCCGTCGCAGTGC**	Padlock probe
PLP-HPV56 E6-1	**AGGCGTCGTGGACGTT**TTCCTTTTACGACCTCAATGCT<br>GCTGCTGTACTACTCTT**CTCACTCAAGTGGTGC**	Padlock probe
PLP-HPV56 E6-2	**ATAAGGAAAATCATCCC**TTCCTTTTACGACCTCAATGCT<br>GCTGCTGTACTACTCTT**CATACTCTGCACACTGC**	Padlock probe
PLP-HPV56 E7-1	**AATTTCTGTTTGAGGTG**TTCCTTTTACGACCTCAATGC<br>TGCTGCTGTACTACTCTT**TCATTGCACTGTAGGTC**	Padlock probe
PLP-HPV56 E7-2	**TGTAAATGTCCAACTGC**TTCCTTTTACGACCTCAATGC<br>TGCTGCTGTACTACTCTT**CAGGTCCTCTTTGGTAC**	Padlock probe
PLP-HPV58 E6-1	**TCAGATCGCTGCAAAG**TTCCTTTTACGACCTCAATGCT<br>GCTGCTGTACTACTCTT**TGTAAAGTCATATACC**	Padlock probe
PLP-HPV58 E6-2	**AACGACCCGAAATATT**TTCCTTTTACGACCTCAATGCT<br>GCTGCTGTACTACTCTT**GCACAGCGCCCTGTCC**	
PLP-HPV58 E6-3	**CGTGGTTTCTCCTCTG**TTCCTTTTACGACCTCAATGCT<br>GCTGCTGTACTACTCTT**CAAATCATGCAATGTC**	Padlock probe
PLP-HPV58 E7-1	**AGAATAGGTCAGTTGG**TTCCTTTTACGACCTCAATGCT<br>GCTGCTGTACTACTCTT**CATAATTGCTCATAGC**	Padlock probe
PLP-HPV58 E7-2	**TGTAGGGTTCGTACGT**TTCCTTTTACGACCTCAATGCT<br>GCTGCTGTACTACTCTT**GCCCATAAGCAGCTGC**	Padlock probe
PLP-HPV58 E7-3	**ATCCTCGTCTGAGCTG**TTCCTTTTACGACCTCAATGCT<br>GCTGCTGTACTACTCTT**CGTCCAAGCCTATTTC**	Padlock probe

PLP-HPV58 E7-4	**GTTGTGCTTGTCCATC**TTCCTTTTACGACCTCAATGCTGCTGCTGTACTACTCTT**TAATTAGCTGTGGCCG**	Padlock probe
PLP-HPV59 E6-1	**AAGTCAGGCAGTTTGT**TTCCTTTTACGACCTCAATGCTGCTGCTGTACTACTCTT**ATTCAATGTTGTGCTC**	Padlock probe
PLP-HPV59 E6-2	**AGTTCCCCTTTGCAAAA**TTCCTTTTACGACCTCAATGCTGCTGCTGTACTACTCTT**ATACCTCTCTTTCTTGC**	Padlock probe
PLP-HPV59 E6-3	**TGCATACGGTGTACAG**TTCCTTTTACGACCTCAATGCTGCTGCTGTACTACTCTT**TGCATTTCAGACACGC**	Padlock probe
PLP-HPV59 E6-4	**ATAGAGGTTTTAGGCA**TTCCTTTTACGACCTCAATGCTGCTGCTGTACTACTCTT**AATTTGTCTGTTGGAC**	Padlock probe
PLP-HPV59 E7-1	**TTTTGGTCCATGCATT**TTCCTTTTACGACCTCAATGCTGCTGCTGTACTACTCTT**TGTCACAAAGTGTTGC**	Padlock probe
PLP-HPV59 E7-2	**TTTTTCATTCTCGGAG**TTCCTTTTACGACCTCAATGCTGCTGCTGTACTACTCTT**CTCCATCTGGTTCATC**	Padlock probe
PLP-HPV59 E7-3	**TGTAAGGCTCGCAATC**TTCCTTTTACGACCTCAATGCTGCTGCTGTACTACTCTT**GTCCATAAACAGCTGC**	Padlock probe
PLP-HPV66 E6-1	**GTGGACGTTCCTGTGT**TTCCTTTTACGACCTCAATGCTGCTGCTGTACTACTCTT**AGATGGTGCAGGCTTC**	Padlock probe
PLP-HPV66 E6-2	**ATATGGCCAATTGT**TTCTTCCTTTTACGACCTCAATGCTGCTGCTGTACTACTCTT**CATACCCTACATACTGC**	Padlock probe
PLP-HPV66 E6-3	**TGCAAACATGACCCGGT**TTCCTTTTACGACCTCAATGCTGCTGCTGTACTACTCTT**TTGTATGTCTCCAACAC**	Padlock probe
PLP-HPV66 E7-1	**AATTTCCGTTTGCGGT**TTCCTTTTACGACCTCAATGCTGCTGCTGTACTACTCTT**CATTGCATTGTAGGTC**	Padlock probe
PLP-HPV66 E7-2	**TAGCTTGCTGTGGCCG**TTCCTTTTACGACCTCAATGCTGCTGCTGTACTACTCTT**TGTTGTTCAGCTTGTC**	Padlock probe
PLP-HPV66 E7-3	**GCTCCTCTTTGGTACTC**TTCCTTTTACGACCTCAATGCTGCTGCTGTACTACTCTT**CTGTTGTACCACACGTA**	Padlock probe
PLP-HPV68 E6-1	**AGGTCTGGCAATTTGT**TTCCTTTTACGACCTCAATGCTGCTGCTGTACTACTCTT**GTCCAATGTCCTGCAC**	Padlock probe
PLP-HPV68 E6-2	**CTGTAAAGTTTCCTGC**TTCCTTTTACGACCTCAATGCTGCTGCTGTACTACTCTT**CAGTGGCGACACTGTC**	Padlock probe
PLP-HPV68 E7-1	**TCTAACACAATTTCCTG**TTCCTTTTACGACCTCAATGCTGCTGCTGTACTACTCTT**CATTGCATGGACATAAC**	Padlock probe
PLP-HPV68 E7-2	**ACGGACACACAAAATT**TTCCTTTTACGACCTCAATGCTGCTGCTGTACTACTCTT**GTTTCCGTTGCACACC**	Padlock probe
Primer	GTAGTACAGCAGCAGCATTGAGG	RCA primer

DP-3-HRP	5' HRP-CCTCAATGCTGCTGCTGTACTAC	HRP labeled detection probe

Supplementary Figures

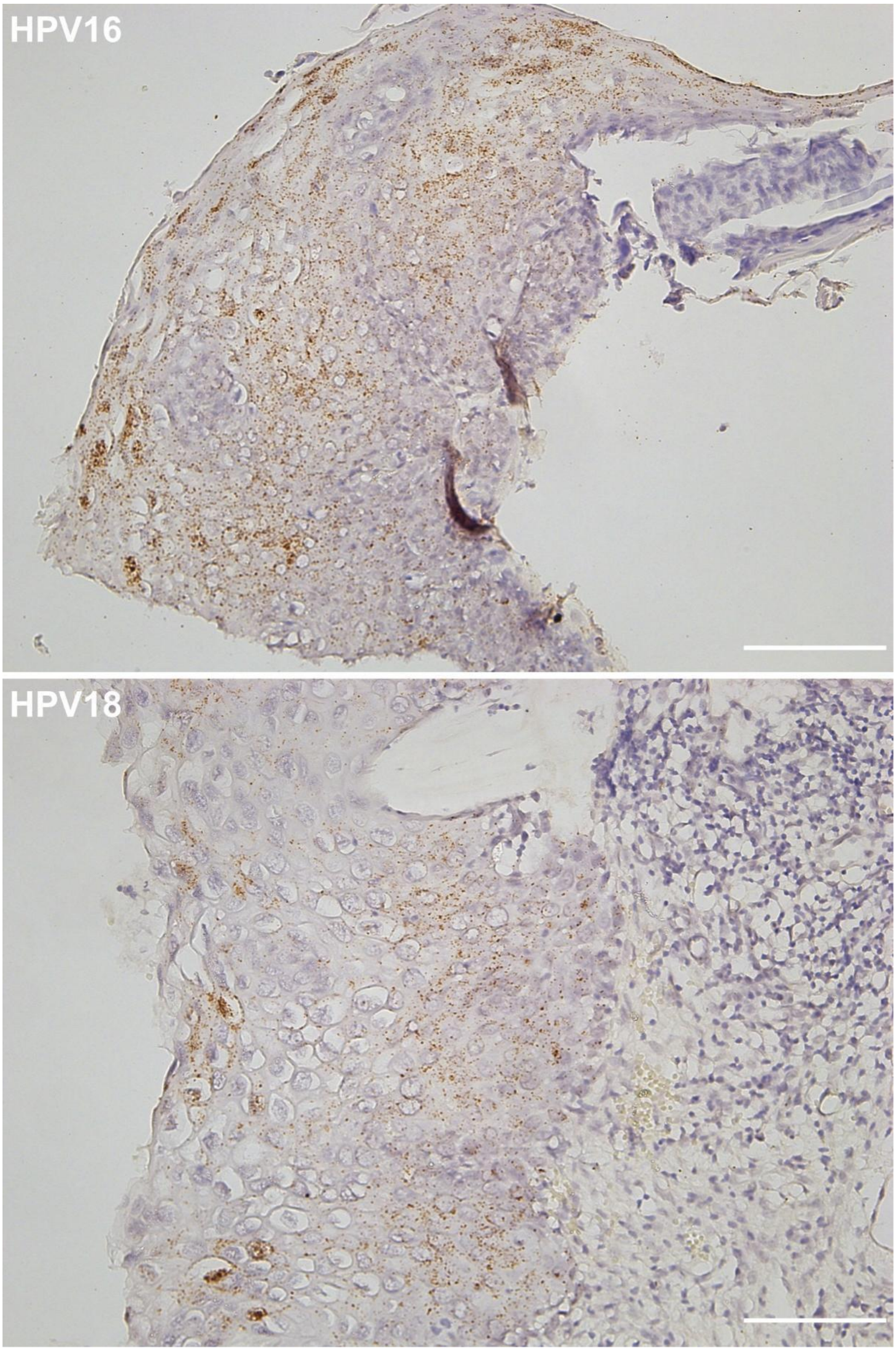

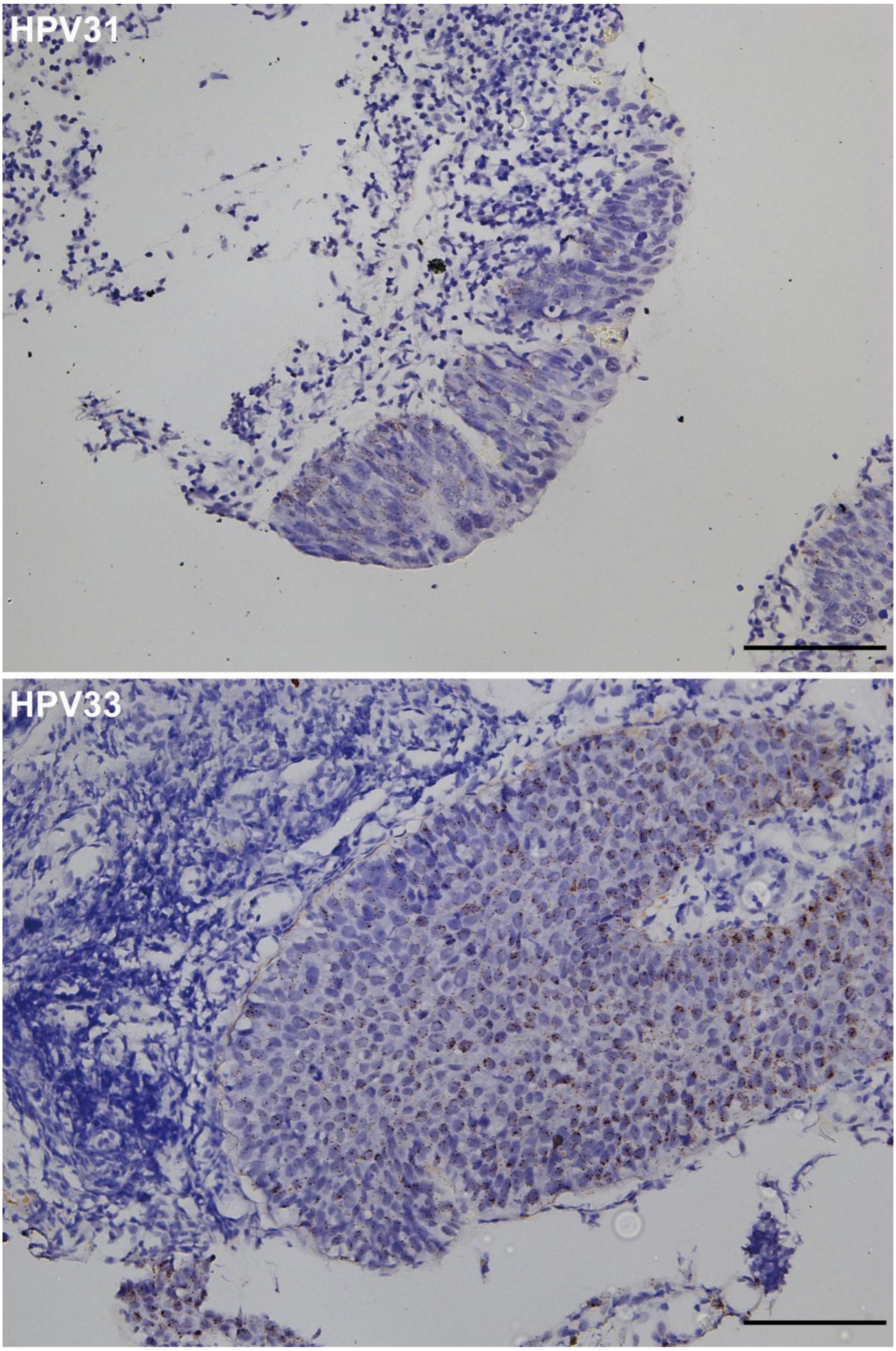
HPV31
HPV33

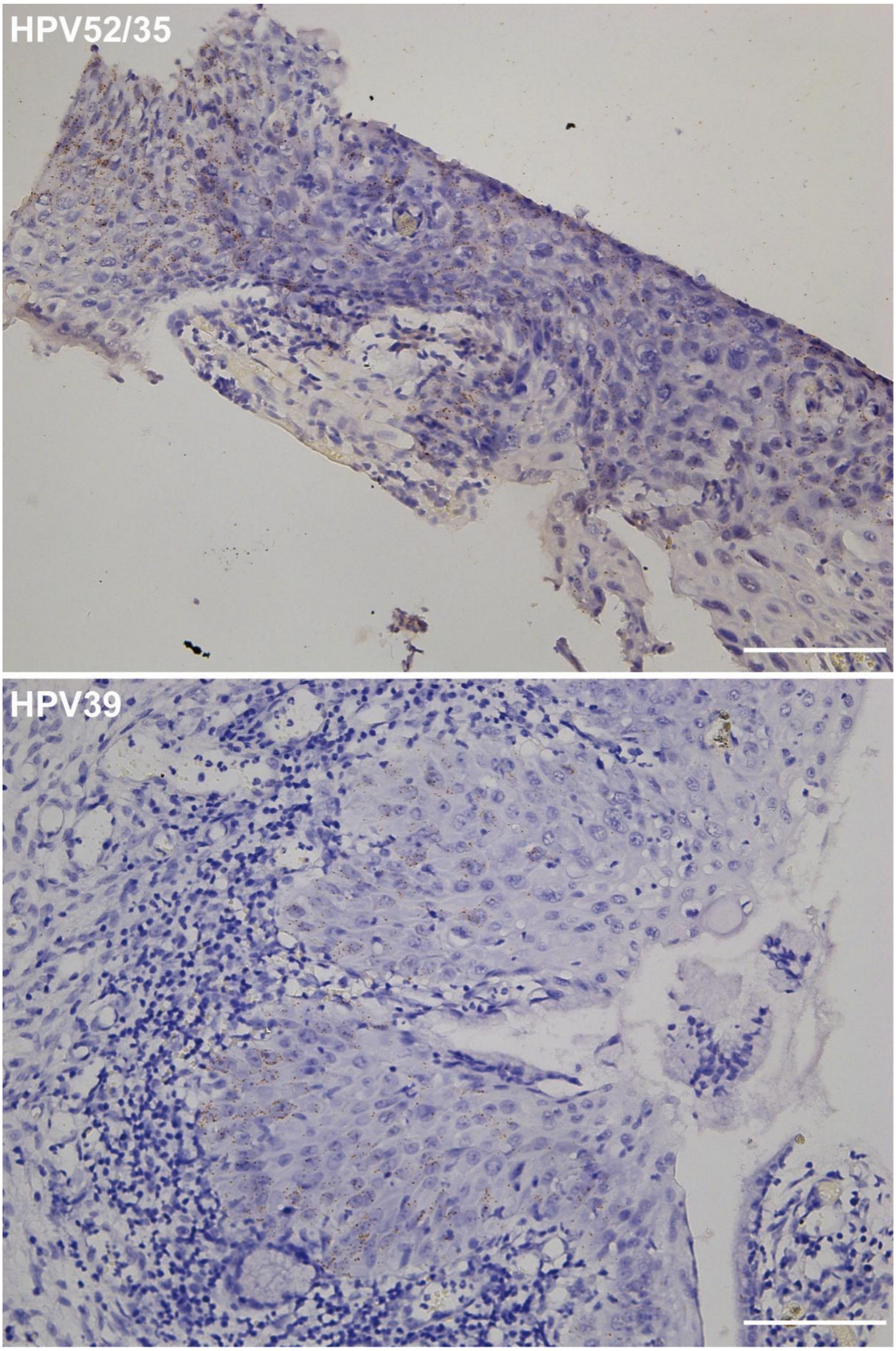
HPV52/35
HPV39

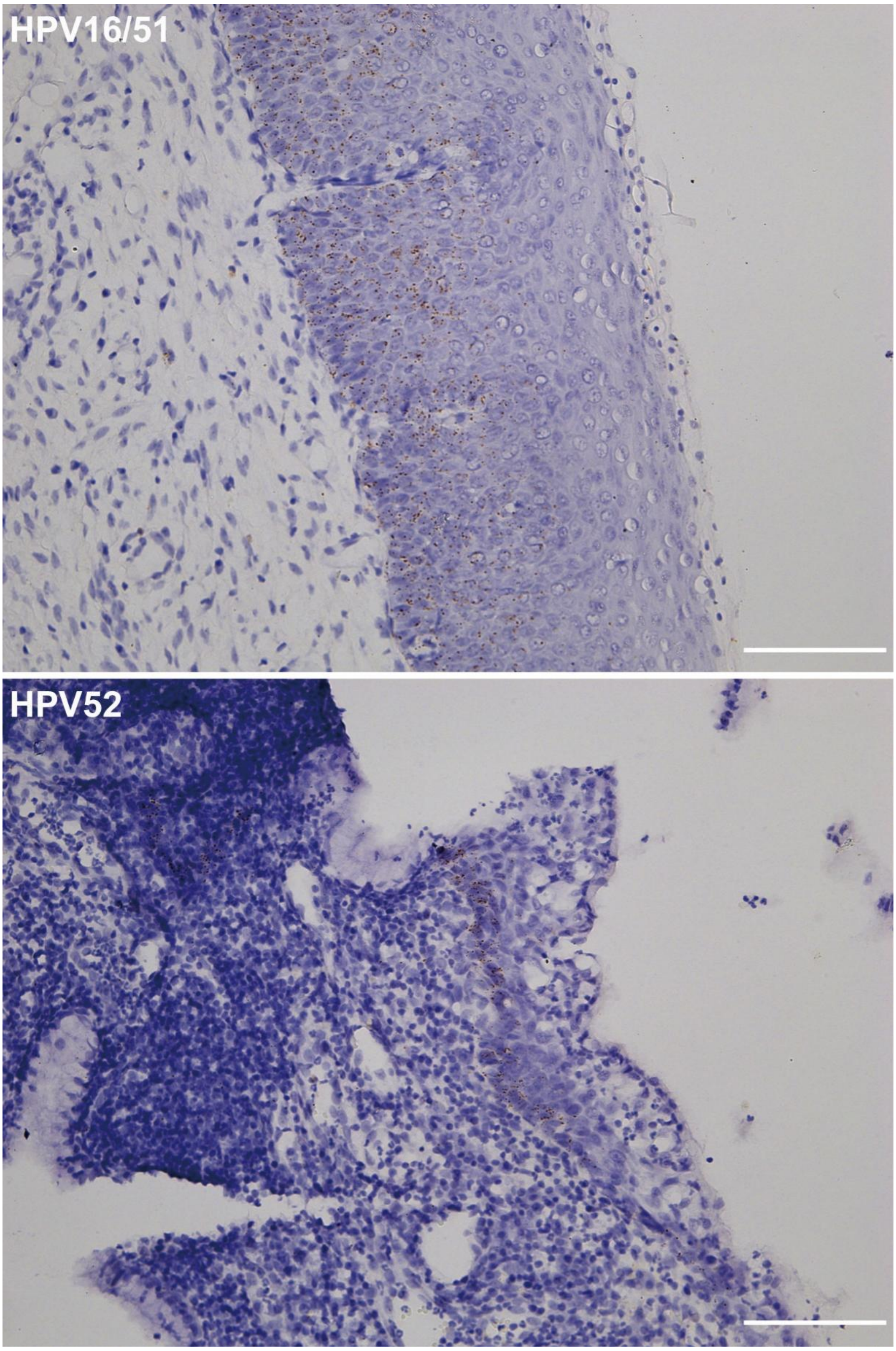
HPV16/51
HPV52

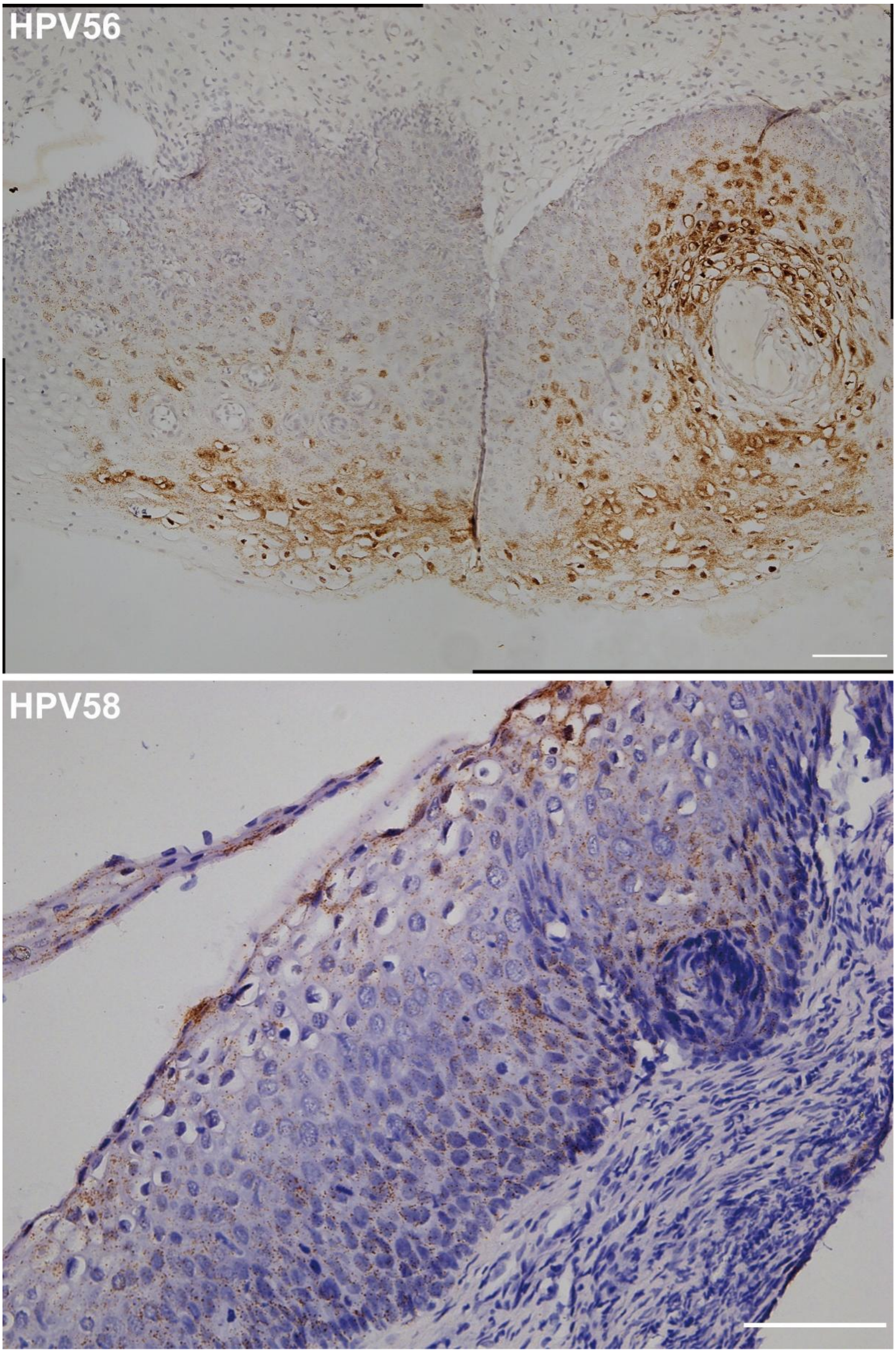
HPV56
HPV58

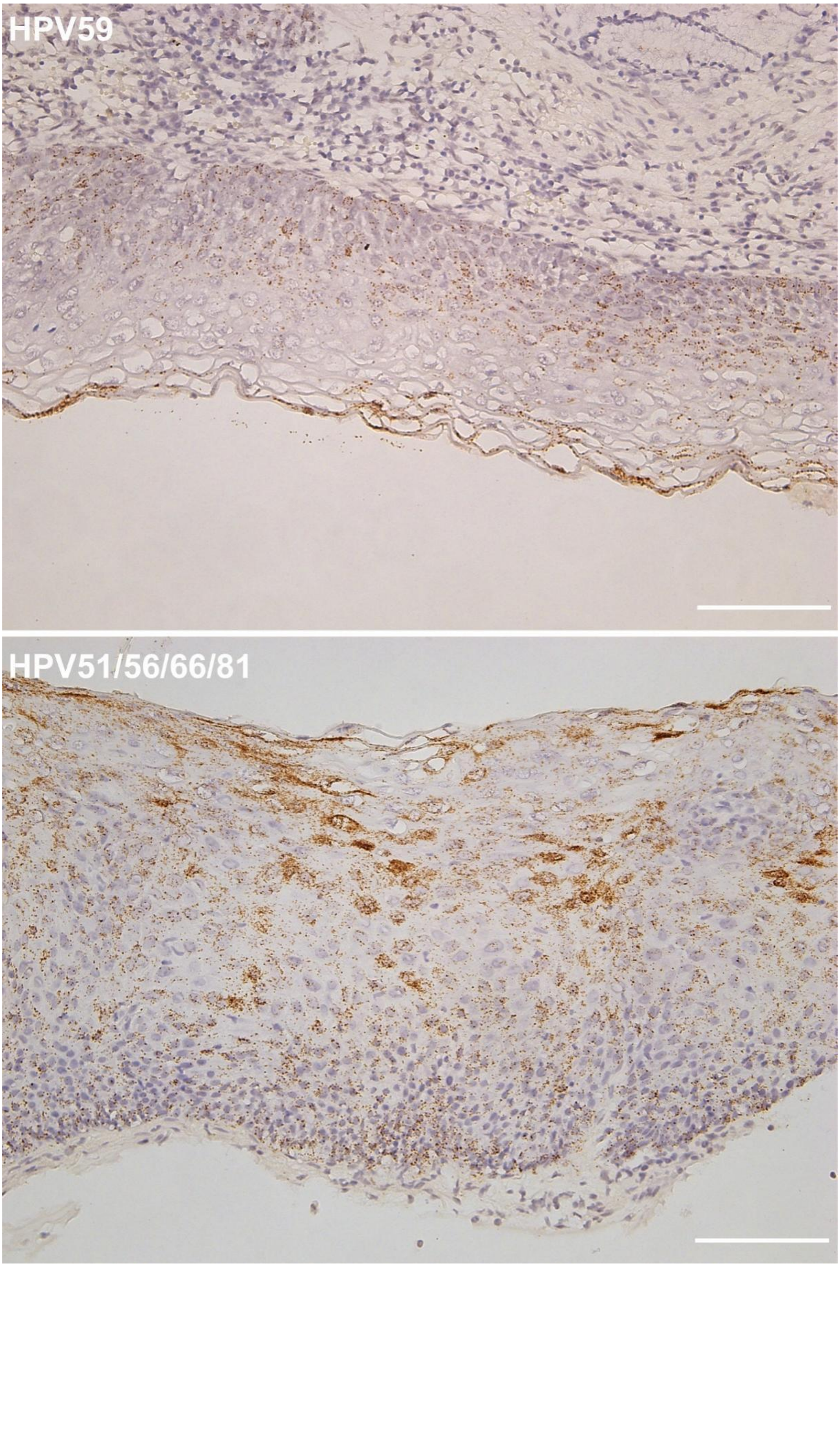
HPV59
HPV51/56/66/81

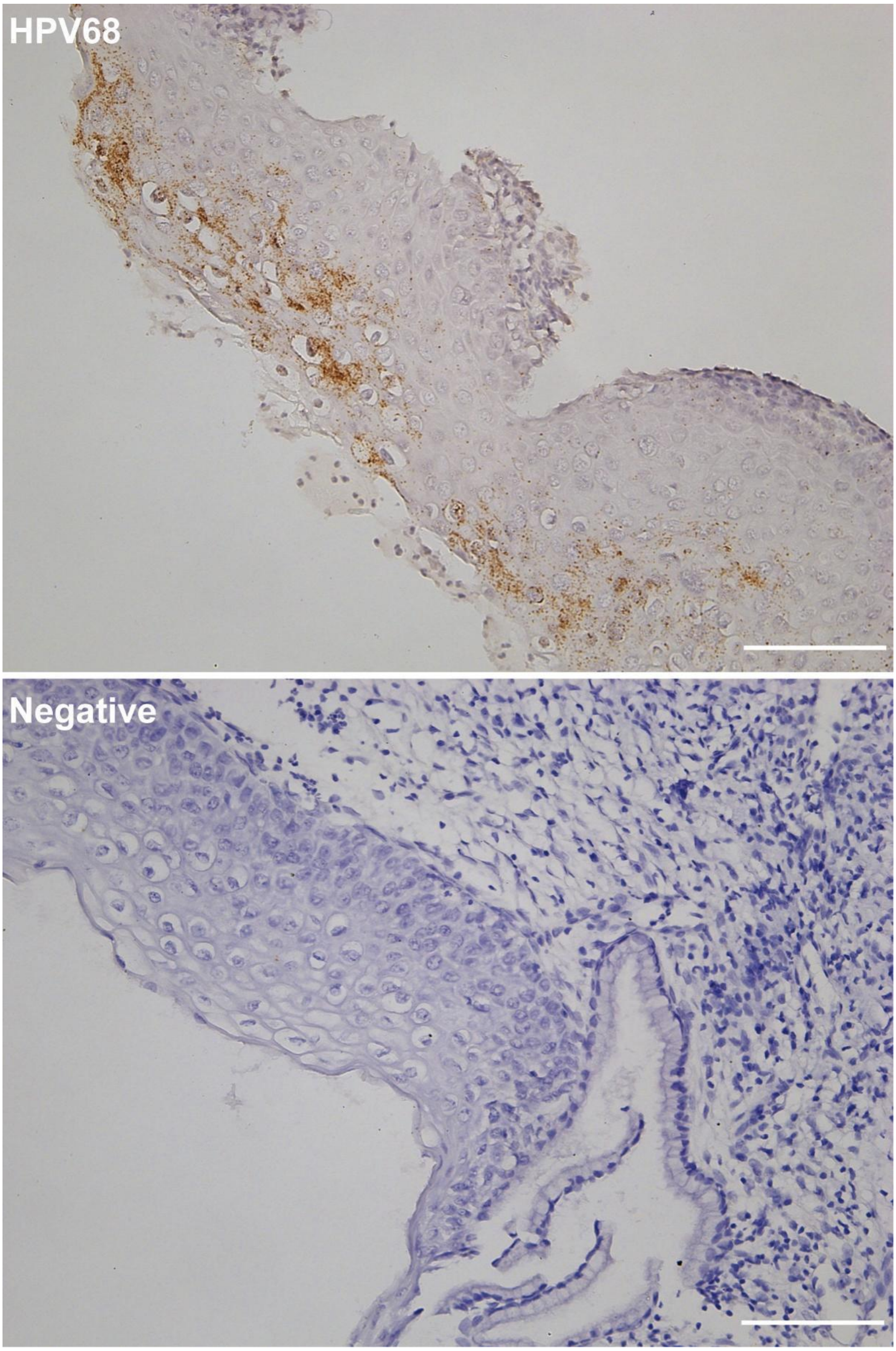

Supplementary Fig 1. HPV E6/E7 mRNA staining in specimens infected with different types of HPV. The negative specimen means non-HPV infection was detected using HPV DNA PCR test in its corresponding cervical swab sample. Blue, nuclei. Brown, E6/E7 mRNA smCISH staining signal. Scale bar, 100 μm.

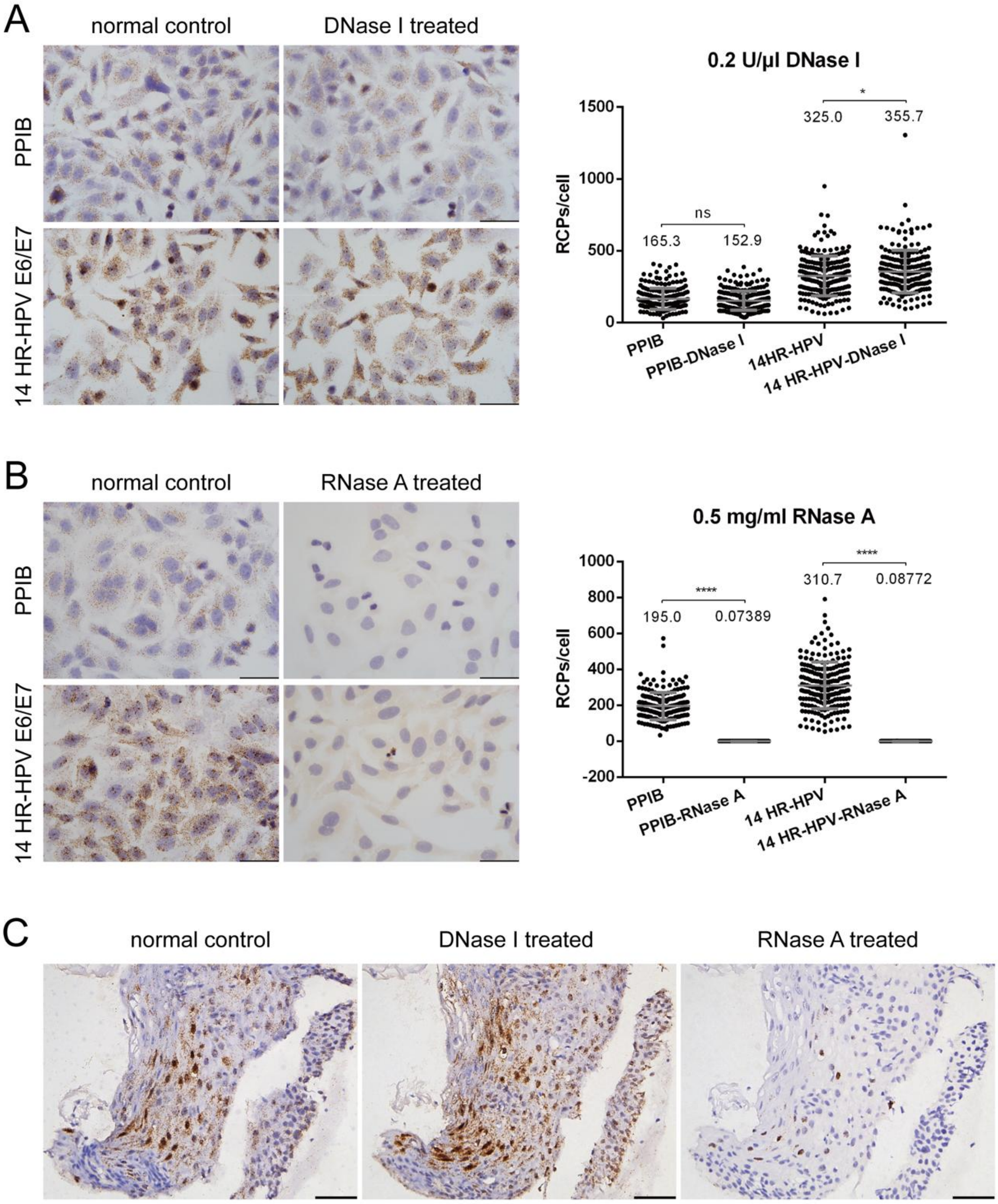

Supplementary Fig 2. Validation of HPV mRNA detection. (A) DNase I treatment showed that there was no signal lost in cell samples. (B) RNase A treatment suggested that the signal detected in cell samples are all mRNA signal because after RNase treatment all signals were lost. It is because RNase has digested the RNA templates, thus no signal can be generated from the padock probes. (C) Tissue control experiment also showed that DNase treatment didn't remove RNA signal. However, the RNase treatment remove the cytoplamic signal but not all nuclei signal, suggesting there may be DNA being detected or RNA. Scale bar: 50 µm.
